# A History of Psychiatric Hospitalization and Surgically Managed Facial Abscess: An Observed-to-Expected Association Analysis

**DOI:** 10.3390/jcm15155910

**Published:** 2026-07-29

**Authors:** Elise Ehland, Amir Maki, Seema Morad, Emma Cornejo

**Affiliations:** 1 Department of Oral and Maxillofacial Surgery, Louisiana State University Health Sciences Center—New Orleans, New Orleans, LA 70119, USA; eehlan@lsuhsc.edu; 2 Department of Psychiatry, Louisiana State University Health Sciences Center—New Orleans, New Orleans, LA 70119, USA; smorad@lsuhsc.edu; 3 School of Dentistry, Louisiana State University Health Sciences Center—New Orleans, New Orleans, LA 70119, USA; ecorn1@lsuhsc.edu

**Keywords:** facial abscess, odontogenic infection, severe mental illness, psychiatric hospitalization, oral health disparities, observed-to-expected ratio

## Abstract

**Background/Objectives**: Severe mental illness is associated with poor oral health, and patients with psychiatric conditions are hospitalized for dental disease more often than the general population. Whether this extends to facial abscesses severe enough to require surgical drainage has not been examined, particularly in United States safety-net populations. We sought to determine whether patients with a history of psychiatric hospitalization are overrepresented among surgically managed facial abscess cases relative to what published general-population rates would predict. **Methods**: Patients were identified at a single tertiary safety-net center between 22 February 2014 and 1 April 2024 using ICD-10 codes for facial abscess together with Epic SlicerDicer psychiatric diagnosis concepts/groups, and cases were confirmed by individual chart review. Observed cases were defined as patients with documented psychiatric hospitalization and documented surgical management of a facial abscess. A denominator of unique psychiatric inpatients over the same interval was obtained through a separate query. Because that query returned an aggregate count without denominator-level follow-up dates, expected cases were estimated by applying published general-population inpatient facial-abscess rates (base case 2.5 per 100,000 person-years; range 2.0–4.0) to approximate person-time, and a crude observed-to-expected ratio with exact Poisson 95% confidence intervals (CIs) was calculated. Temporal ordering of abscess and psychiatric admission was examined where sufficient timing data were available. **Results**: Eighty patients met the case definition, against 3.37 expected under the base-case rate, giving a crude observed-to-expected ratio of 23.8 (95% CI 18.9–29.6). The ratio ranged from 14.9 to 29.7 across the reference-rate range. Among the 67 cases with sufficient timing data, 30 (45%) preceded the first psychiatric admission, 1 (1%) occurred during a psychiatric hospitalization, and 36 (54%) followed discharge, so the finding is presented as an association rather than an incidence. The abscess was odontogenic in 45 cases (56%), non-odontogenic in 19 (24%), and unspecified in 16 (20%). Substance use disorders (32.5%) and schizophrenia-spectrum disorders (32.5%) were most frequently represented, with a mean of 2.8 psychiatric admissions per patient. **Conclusions**: In this single-center safety-net dataset, patients with a documented history of psychiatric hospitalization were substantially overrepresented among surgically managed facial abscess cases relative to published population rates. Because the analysis is a crude observed-to-expected comparison without dynamic denominator-level person-time or demographic standardization, and because a substantial minority of abscesses preceded the psychiatric admission, the ratio reflects the combined burden of psychiatric hospitalization and its associated exposures rather than an independent causal effect. Patient-level linked cohort studies are needed to estimate the magnitude of risk reliably.

## 1. Introduction

Facial abscesses are localized collections of pus within the soft tissues of the face, and the great majority arise from an odontogenic source. Dental caries and periodontal disease seed infection into the periapical tissues, from which pus erodes through cortical bone and enters the surrounding fascial planes [1]. The route it takes is determined by the anatomy of the offending tooth. Infection from the mandibular molars, whose roots lie below the attachment of the mylohyoid muscle, passes readily into the submandibular space and from there to the sublingual and submental spaces, producing the bilateral floor-of-mouth cellulitis known as Ludwig angina, in which the tongue is displaced upward and backward and the airway is progressively obstructed [2,3]. Spread may continue into the submasseteric, pterygomandibular, lateral pharyngeal, and retropharyngeal spaces, encircling the airway, and can descend along the cervical fascia into the mediastinum [4]. The recognized complications include airway obstruction, descending necrotizing mediastinitis, cavernous sinus thrombosis, cerebral abscess, necrotizing fasciitis, and death [1,5]. Non-odontogenic sources, including trauma, infected fixation hardware, and salivary gland obstruction, account for a smaller but clinically important share.

Most facial abscesses never reach this point, and are managed in the outpatient setting with antibiotics and timely dental treatment. A minority progress to deep fascial-space infection requiring urgent surgical drainage and hospital admission. Abscess is the most common principal diagnosis among dental-related inpatient admissions in the United States [6,7], and national inpatient and emergency-department data place the general-population rate of hospitalization for these infections on the order of a few cases per 100,000 persons each year [8]. Hospital series consistently identify poor oral hygiene, absent routine dental care, and systemic comorbidity as antecedents, and diabetes mellitus in particular is associated with deep neck infection and more aggressive spread of inflammation [9].

Severe facial abscess often represents the end result of oral disease that has not been prevented or treated early. For that reason, populations with persistent barriers to routine dental care may be disproportionately represented among these infections. Patients with severe mental illness are one such population. A meta-analysis of 25 studies found markedly higher rates of total tooth loss and dental caries in this group than in the general population [10]. Register-based work also reported an age- and sex-standardized admission ratio of approximately 2.55 for dental caries as a cause of repeated hospital admission, with substance use, smoking, diabetes, and depressive disorder emerging as independent predictors [11]. Oral health is increasingly recognized as a neglected component of psychiatric care [12,13], and the population affected is large: national survey data indicate that tens of millions of adults in the United States live with a mental illness or substance use disorder each year [14]. What remains less clear is whether these oral-health disparities extend to the severe end of the spectrum, where infection progresses to abscess formation and procedural drainage.

Several mechanisms make that extension plausible. Psychotropic medications, including tricyclic antidepressants, second-generation antidepressants, antipsychotics, and the anticholinergic agents prescribed alongside them, reduce salivary flow by blocking muscarinic receptors in the salivary glands; a meta-analysis of second-generation antidepressants confirmed the elevated risk of dry mouth [15], and reported prevalences of xerostomia across psychiatric drug classes range from roughly ten to sixty-five percent depending on the agent [16]. Saliva buffers acid, clears substrate, and delivers antimicrobial proteins, so its loss accelerates caries and periodontal breakdown in precisely the tissues from which odontogenic infection arises. Acting alongside these pharmacological effects are reduced motivation for daily oral hygiene, financial and transport barriers, competing clinical priorities during psychiatric crises, and difficulty tolerating dental treatment. Each of these pathways would be expected to raise the likelihood that dental disease is neither prevented nor treated, and therefore that it progresses toward the complications described above [17].

Despite these biologically and clinically plausible links, the burden of surgically managed facial abscess among patients with psychiatric hospitalization has not been well quantified. Much of the existing literature comes from national health systems that differ from United States safety-net hospitals in payer mix, outpatient dental access, and patterns of acute care use. Case series of deep neck infection have identified depressive, schizoaffective, schizophrenic, and bipolar disorders among possible predisposing conditions, but have not examined the psychiatric inpatient population as the source population [18]. We therefore compared the number of surgically managed facial abscess cases observed among patients with documented psychiatric hospitalization at a single safety-net institution with the number expected from published general-population rates. Because the dataset was assembled retrospectively around the outcome and did not include an internal unexposed comparison group, the most defensible design available to us was an observed-to-expected comparison rather than a cohort or case–control study.

Clinical series of severe odontogenic infection also support using anatomic-space involvement and procedural drainage as markers of clinically meaningful disease. Hospitalized odontogenic infections are commonly described by fascial-space involvement, trismus, dysphagia, need for incision and drainage, and length of stay rather than by the physical location where drainage occurs [19]. Reviews of odontogenic infection complications similarly emphasize that delayed source control can allow spread into deep cervical spaces, the airway, mediastinum, orbit, cavernous sinus, and intracranial compartments [20]. This distinction is important for the present study because the captured infections represent advanced facial-space disease rather than routine dental caries or uncomplicated periapical infection.

## 2. Materials and Methods

### 2.1. Study Design and Identification of Cases

This study was a retrospective observed-to-expected analysis conducted at a single tertiary safety-net center. Candidate patients were identified using the Epic SlicerDicer self-service analytics tool by querying for the co-occurrence of International Classification of Diseases, Tenth Revision (ICD-10) diagnosis codes for facial abscess and Epic SlicerDicer psychiatric diagnosis concepts/groups over the interval 22 February 2014 to 1 April 2024. Facial abscess was defined by two ICD-10 codes: K12.2, cellulitis and abscess of mouth, which encompasses cellulitis of the floor of the mouth, submandibular abscess, and Ludwig angina; and J39.1, another abscess of the pharynx, which encompasses parapharyngeal abscess. These codes capture infection that has spread beyond the tooth into the surrounding soft tissue and fascial spaces. Under ICD-10 conventions both carry Excludes2 notes for periapical abscess (K04.6–K04.7) and periodontal abscess (K05.21), meaning that those entities are not themselves represented by K12.2 or J39.1 although a patient may carry both a periapical and a cellulitis code concurrently. The implications of this coding structure for comparability with the published reference rates are considered in the Section Limitations. Because the presence of a diagnosis code or Epic diagnosis concept does not by itself confirm the clinical presence or severity of disease, every candidate chart was reviewed individually rather than accepted on the basis of coding alone.

Psychiatric diagnoses were identified using Epic SlicerDicer diagnosis concepts/groups selected from the institutional Epic diagnosis hierarchy rather than a manually curated list of individual ICD-10 psychiatric codes. The selected concepts included schizophrenia and schizophrenia-spectrum disorders, schizoaffective disorder, schizophreniform disorder, bipolar disorder, major depressive disorder, generalized anxiety disorder, and substance abuse, substance dependence, and substance use disorder concepts. During chart review, psychiatric diagnoses were then grouped into clinically interpretable categories for reporting: substance use disorder, schizophrenia-spectrum/psychotic disorders, depressive disorders, bipolar disorder, and anxiety/trauma-related disorders.

**Figure 1:**
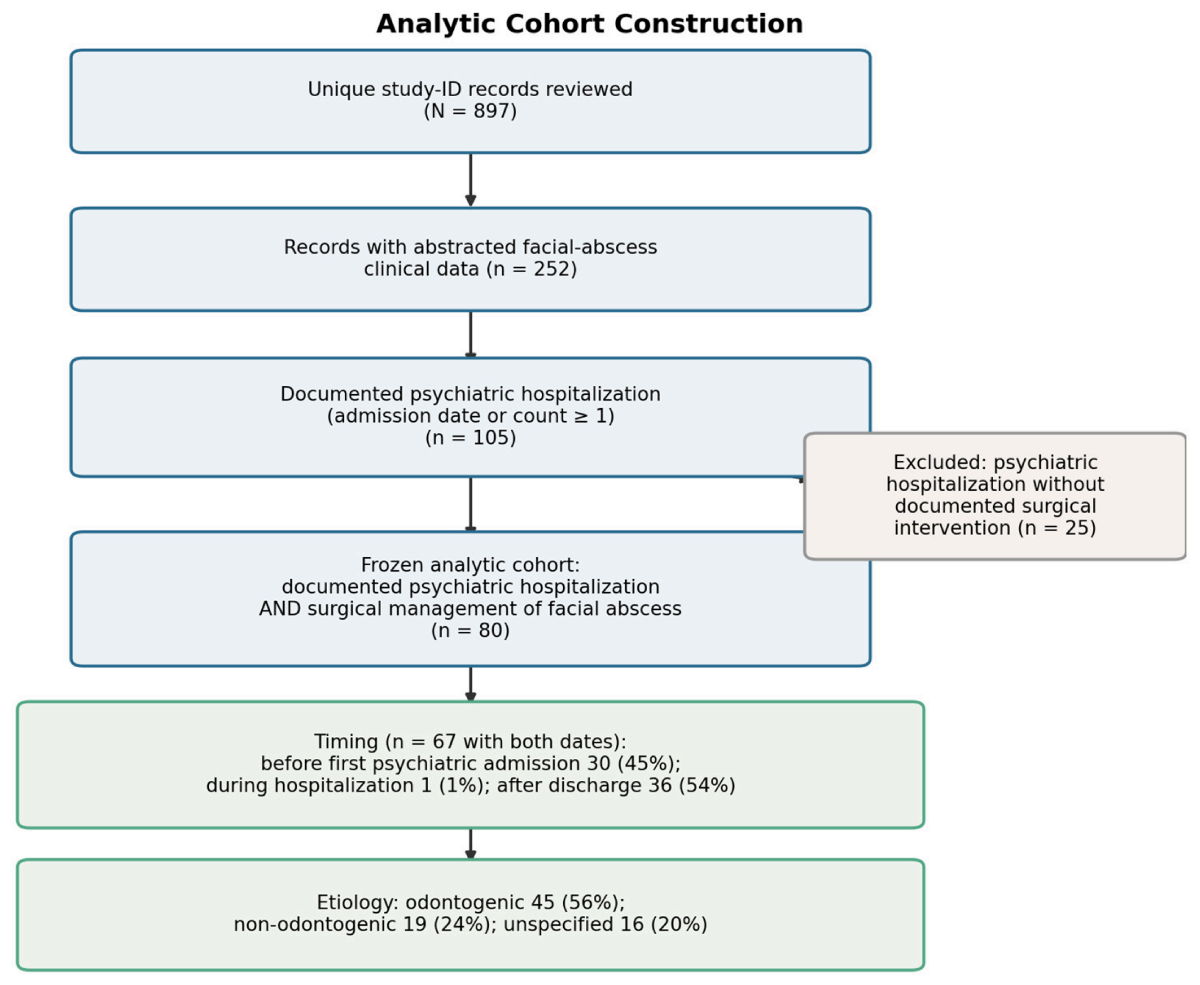
Construction of the analytic cohort. Of 897 unique records reviewed, 252 had abstracted facial-abscess data and 105 a documented psychiatric hospitalization; 25 were excluded for absence of documented surgical management, yielding 80 observed cases. Temporal and etiological distributions are shown beneath.

Manual review confirmed 252 patients with abstracted facial-abscess clinical data, of whom 105 had a documented psychiatric hospitalization. Observed cases were defined by a single explicit rule: documentary evidence of both a psychiatric hospitalization (a recorded psychiatric admission date or a documented admission count of one or more) and surgical management of the facial abscess (a recorded operative date or a documented intervention count of one or more). Applying this rule identified 80 observed cases; 25 psychiatrically hospitalized patients in whom surgical management could not be verified were excluded. The construction of the analytic cohort is shown in Figure 1. Surgical management was defined as any procedure requiring a deliberate incision to drain an abscess collection. The cohort was not restricted to the operating room: bedside incision and drainage performed in the emergency department or on the ward was included wherever drainage was documented. Antibiotic therapy alone, dental extraction alone, or a diagnosis code without documented drainage did not qualify. Procedure setting was not treated as a marker of severity, since at our institution submandibular and other facial-space abscesses are routinely drained at the bedside by the oral and maxillofacial surgery service although comparable infections are taken to the operating room elsewhere; severity is instead described using anatomic space involvement where available, recurrence, need for repeat drainage, and length of stay. This cohort was re-audited against the complete abstraction dataset during revision, which refined the count from a preliminary figure of 82 to the frozen value of 80.

During the preparation of this manuscript, the authors used Claude Opus 4.5 (Anthropic, PBC, San Francisco, CA, USA; https://www.anthropic.com) for proof-reading, and formatting assistance. The authors have reviewed and verified all output and take full responsibility for the content of this publication.

### 2.2. Denominator and Person-Time

The denominator was obtained through a separate SlicerDicer query returning the total number of unique patients admitted to psychiatry at the same institution over the identical interval, without any facial-abscess restriction. This query returned 13,322 unique patients as an aggregate count. The numerator and denominator were defined using the same institutional Epic/SlicerDicer psychiatric-hospitalization criteria and the same study interval; therefore, patients included in the observed cohort met the eligibility definition for the psychiatric-hospitalized denominator population, even if the facial abscess was treated during a later surgical or medical admission. The denominator query, however, returned an aggregate count rather than a full patient-level follow-up file with index admission dates, death dates, or last-contact dates for every psychiatric inpatient. In the absence of individual follow-up times for the full denominator, approximate person-time was calculated as the product of the unique patient count and the study duration of 10.1 years. This approximation almost certainly overestimates true person-time, since patients entering late in the interval could not have contributed the full duration; among the observed cases for whom admission dates were recoverable, the mean interval from first psychiatric admission to the study end date was approximately 5.7 years. The person-time assumption is therefore treated as a primary limitation rather than a settled input and is emphasized in the Limitations. Psychiatric inpatient admission at this institution occurs predominantly through the emergency department; any psychiatric admissions arriving by direct transfer that were not captured by the denominator query would reduce the denominator, lower the expected count, and therefore inflate the observed-to-expected ratio, biasing the estimate away from the null.

### 2.3. Estimation of Expected Cases and Statistical Analysis

Expected cases were estimated by applying a published general-population rate of inpatient facial abscess to the approximate person-time of the psychiatric population. National inpatient and emergency-department datasets indicate a general-population rate of roughly 2 to 4 inpatient facial-abscess cases per 100,000 person-years [6,7,8]; we adopted a base-case rate of 2.5 per 100,000 person-years and examined 2.0 and 4.0 as alternatives. Because the reference rates are not available stratified by age and sex at a granularity matching our cohort, no demographic standardization was possible; the resulting estimate is therefore a crude observed-to-expected ratio rather than a standardized incidence ratio. The ratio was calculated as observed divided by expected cases, with exact 95% confidence intervals (CIs) derived from the Poisson distribution of the observed count. These intervals reflect variability in the observed count alone and treat the reference rate as fixed; they therefore understate total uncertainty, which the reference-rate range is intended to bracket only in part. Where sufficient timing data were available, the temporal relationship was determined from the psychiatric admission date, recorded hospitalization duration, and abscess event date. Descriptive statistics characterized the cohort, and missingness is reported for each key variable. All analyses were performed in Python (version 3.12; pandas and SciPy libraries).

## 3. Results

### 3.1. Observed-to-Expected Ratio

**Figure 2:**
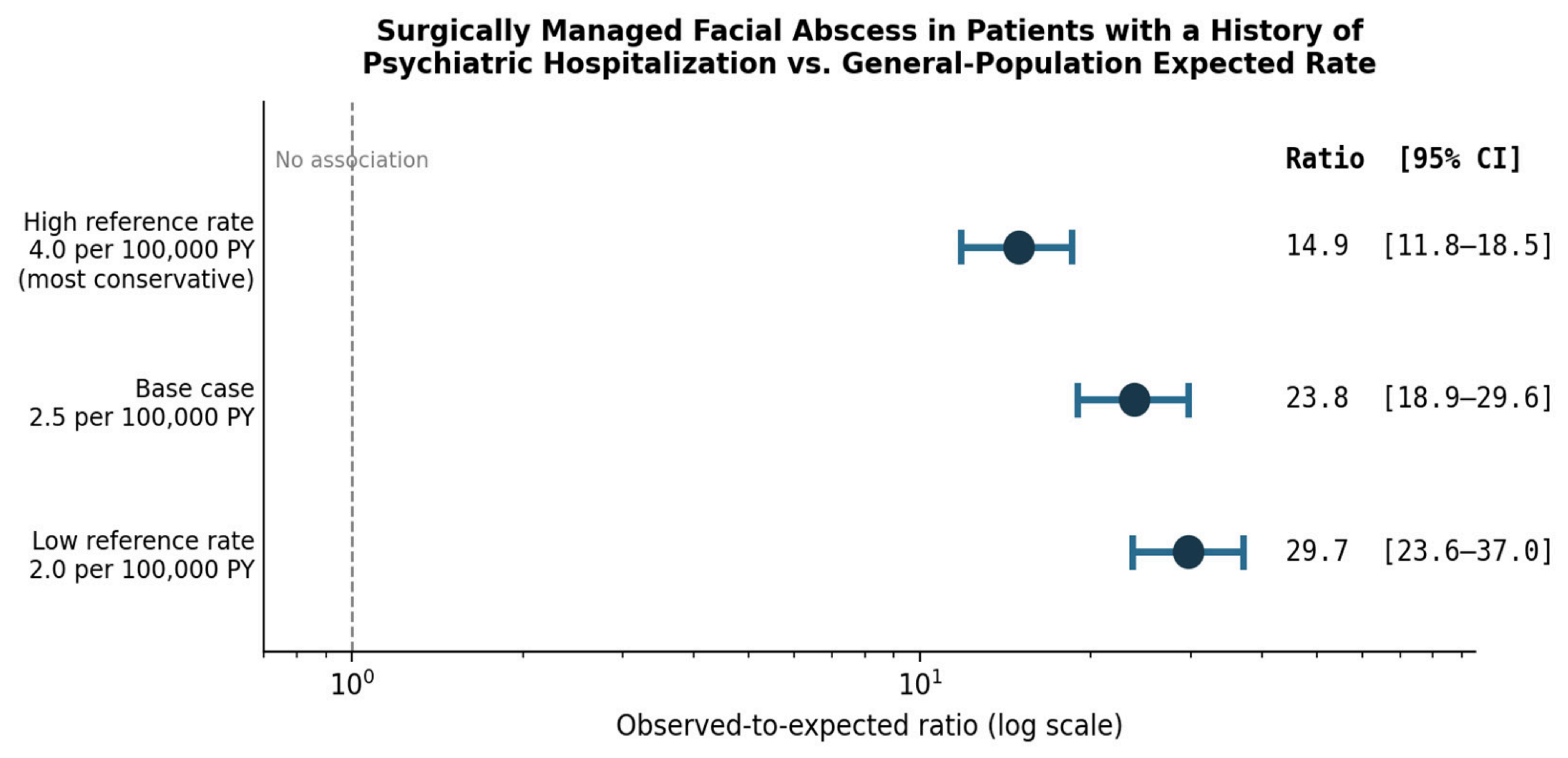
Crude observed-to-expected ratio of surgically managed facial abscess in patients with a history of psychiatric hospitalization, relative to the general-population expected rate, across reference-rate assumptions on a logarithmic scale. A higher reference rate yields a lower, more conservative ratio. Confidence intervals reflect Poisson variability in the observed count and treat the reference rate as fixed.

Eighty patients met the case definition. Against an approximate person-time of 134,619 person-years and the base-case general-population rate of 2.5 per 100,000 person-years, 3.37 cases would have been expected, giving a crude observed-to-expected ratio of 23.8 (95% CI 18.9–29.6). The ratio was sensitive to the assumed reference rate in the expected direction: a higher reference rate of 4.0 per 100,000 person-years, the most conservative assumption, produced a ratio of 14.9 (95% CI 11.8–18.5), while a lower rate of 2.0 produced a ratio of 29.7 (95% CI 23.6–37.0) (Table 1; Figure 2). Because the confidence intervals reflect Poisson variability in the observed count alone and treat the reference rate as fixed, and because the person-time is approximate, these values are best read as indicating a large overrepresentation of surgically managed facial abscess in this population rather than as a precise measure of its magnitude.

### 3.2. Characteristics of Affected Patients

**Figure 3:**
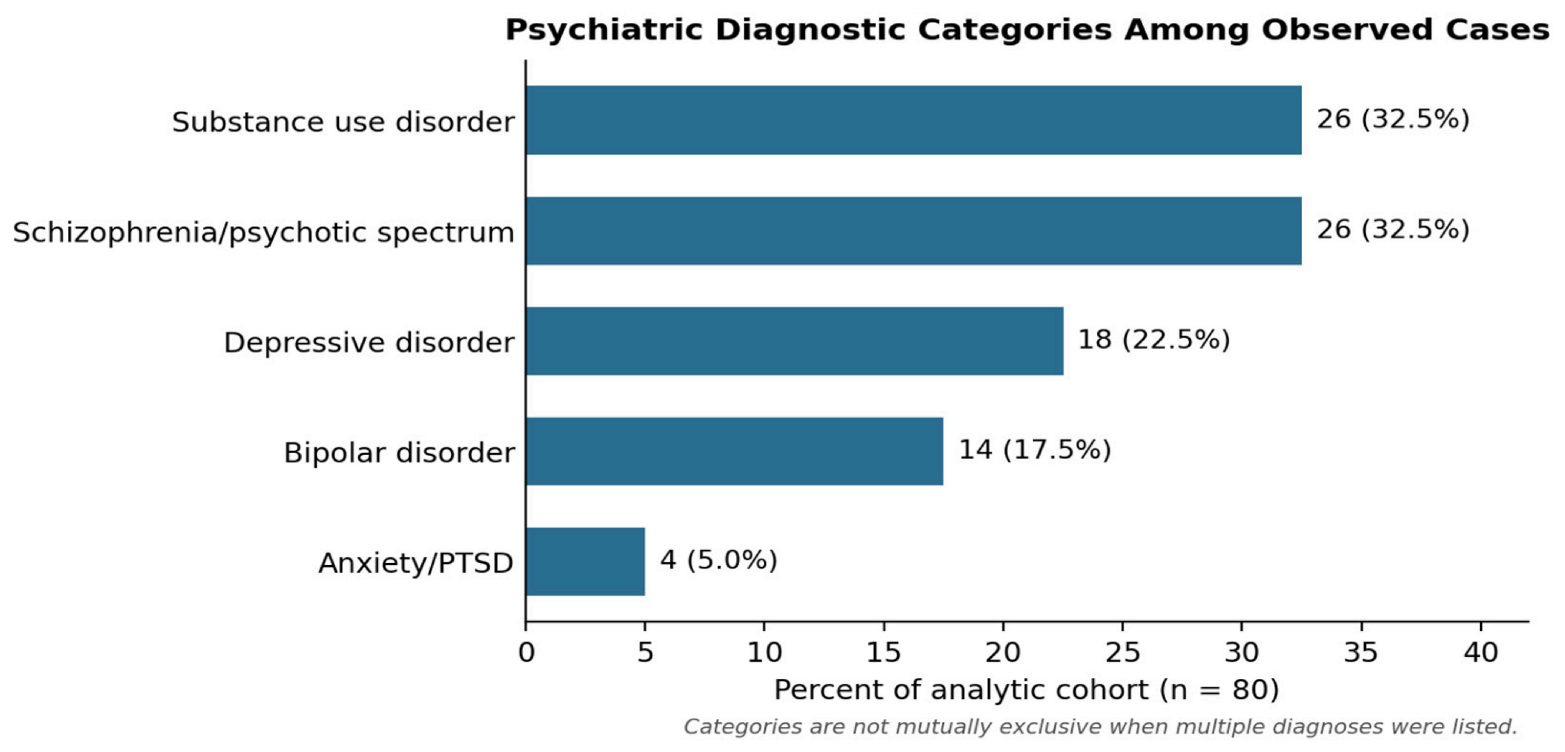
Psychiatric diagnostic categories among the 80 observed cases, expressed as a percentage of the analytic cohort. Categories are not mutually exclusive, as some patients carried more than one documented diagnosis. PTSD, post-traumatic stress disorder.

The 80 patients had a mean age of 45.9 ± 12.1 years (range 21–70) and were predominantly male (61.3%, *n* = 49). Consistent with the safety-net catchment of the institution, the population was largely Black/African American (64.6% of known race) and White/Caucasian (31.6%); race was unrecorded in one case. The psychiatric burden was considerable: patients had a mean of 2.8 psychiatric admissions (median 2, maximum 17), with 221 admissions in aggregate. The most frequently represented diagnostic categories were substance use disorders (32.5%) and schizophrenia-spectrum and other psychotic disorders (32.5%), followed by depressive disorders (22.5%), bipolar disorder (17.5%), and anxiety or trauma-related disorders (5.0%) (Figure 3); categories are not mutually exclusive, since many patients carried more than one documented diagnosis. For reporting, substance use disorder included Epic/chart-documented substance abuse, substance dependence, or substance use disorder concepts, including alcohol, opioid, stimulant/cocaine/methamphetamine, cannabis, sedative, or polysubstance diagnoses when present; isolated social use without documentation of a disorder was not counted. Documented diabetes was present in five patients (6%), smoking in 16 (20%), and substance or alcohol use in 31 (39%); these are counts of positive documentation and therefore represent lower bounds.

The facial-abscess course was frequently complicated. More than one surgical intervention was required in 26.2% of patients (21 of 80), and the abscess recurred in 20.5% of those with a documented recurrence status (15 of 73). Length of stay for the facial-abscess admission was documented in 76 of 80 cases. Because recurrence counts had been entered inconsistently by the several personnel who performed data abstraction, recurrence was analyzed only as a binary variable; missingness for each key variable is reported in the Section Limitations.

### 3.3. Temporal Ordering and Etiology

**Figure 4:**
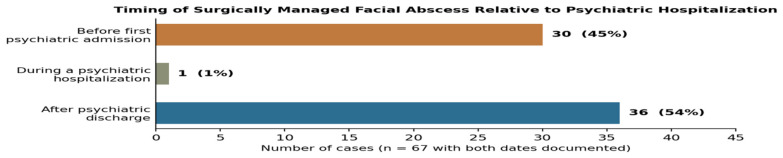
Timing of surgically managed facial abscess relative to psychiatric hospitalization, among the 67 cases with sufficient timing data. Classification was based on the documented psychiatric admission date, recorded duration of psychiatric hospitalization, and abscess event date.

Admission and event dates were used to establish the temporal relationship between psychiatric hospitalization and facial abscess. Timing was established from the documented psychiatric admission date, the recorded duration of psychiatric hospitalization, and the abscess event date, with the discharge date derived from admission date and duration when necessary. Each abscess was classified as preceding the first psychiatric admission, occurring during a psychiatric hospitalization, or following psychiatric discharge. Among the 67 cases with sufficient timing data, 30 (45%) preceded the first psychiatric admission, one (1%) occurred during a psychiatric hospitalization, and 36 (54%) followed discharge (Figure 4). In the remaining 13 cases, psychiatric hospitalization was documented by count but a recoverable admission date was not available. Because a substantial minority of abscesses preceded the psychiatric admission, the analysis is presented throughout as an association between a history of psychiatric hospitalization and surgically managed facial abscess, rather than as an incidence of abscess following psychiatric hospitalization.

The cause of the facial abscess was odontogenic in 45 cases (56%), non-odontogenic in 19 (24%), and unspecified in 16 (20%). The non-odontogenic cases comprised infected fixation hardware, post-traumatic infection, gunshot injury, and salivary gland infection. We suspect that most unspecified cases were odontogenic, since abstraction of the cause field was inconsistent, but this cannot be demonstrated and has not been assumed. The findings therefore apply to surgically managed facial abscess of predominantly, though not exclusively, odontogenic origin. Anatomic space involvement was recorded in the same free-text field and was available in only 9 of the 80 cases. Where it was documented, the infections involved the submandibular, buccal, pterygomandibular, lateral pharyngeal, retropharyngeal, submasseteric, and deep and superficial temporal spaces, as well as the maxillary vestibule and hard palate, and one case recorded simultaneous involvement of seven spaces. These entries confirm that the disease captured here reaches the deep fascial spaces, but because the field was not completed systematically the distribution is reported descriptively and was not used as a severity variable.

## 4. Discussion

In this single-center analysis, patients with a documented history of psychiatric hospitalization were overrepresented among surgically managed facial abscess cases compared with what would be expected under published general-population rates. The base-case crude observed-to-expected ratio was 23.8, with a range of 14.9 to 29.7 across the reference-rate assumptions examined. To our knowledge, this is the first study to examine the burden of surgically managed facial abscess in relation to psychiatric hospitalization in a United States safety-net setting. Because the analysis is crude and relies on approximate person-time and external reference rates, it should be read as evidence of a strong association, not as a precise measure of risk.

These findings fit within a broader literature linking severe mental illness with poor oral health. A meta-analysis of 25 studies found substantially higher rates of tooth loss and caries in this population [10], and register-based work reported an age- and sex-standardized admission ratio of approximately 2.55 for dental caries as a cause of repeated hospital admission [11]. Our observed-to-expected ratio is larger, but the two estimates should not be compared directly. They differ in endpoint, baseline rate, case definition, population, and ascertainment method, and any one of these factors could change the magnitude of a relative estimate. Our endpoint captures procedurally managed disease at the severe end of a spectrum whose milder forms are usually handled in outpatient dental settings, while our safety-net cohort also concentrates tobacco use, substance use, and access barriers that often accompany severe mental illness. These factors make a large association plausible, but they do not establish its exact size.

Substance use disorder is particularly relevant to this association. Meta-analytic data show that patients with substance use disorders have greater and more severe caries and periodontal disease and are less likely to have received dental care than the general population [21]. This may reflect multiple converging mechanisms, including xerostomia, high-sugar intake, bruxism, direct mucosal exposure to inhaled or ingested substances, tobacco use, poor access to preventive dental services, and difficulty completing staged dental treatment [22]. A separate systematic review and meta-analysis focused on people who use drugs likewise found a higher burden of caries and periodontal disease, supporting substance use as a clinically important cofactor rather than a simple comorbidity in this population [23].

Periodontal deterioration provides another biologically plausible pathway. Severe mental illness has been associated with advanced dental disease, tooth loss, and higher decayed–missing–filled indices [24], and patients with chronic schizophrenia have been described as carrying a neglected oral-health burden requiring structured assessment, management of dry mouth, and early dental referral [25]. Medication effects may compound these behavioral and access-related risks: psychotropic medications used for depression, psychosis, anxiety, and mood disorders have well-described oral adverse effects, particularly xerostomia, which reduces salivary buffering and clearance and may accelerate caries, gingival inflammation, and periodontal breakdown [26]. Population-level work also supports an association between mental health status and oral-health status and care utilization, reinforcing that the pathway is likely social, behavioral, pharmacologic, and biological rather than purely psychiatric [27].

The clinical implication is best framed as targeted vigilance rather than proof of causation. A history of psychiatric hospitalization identifies a group in which surgically managed facial abscess was markedly overrepresented, and the observed recurrence and repeat-drainage rates suggest that these infections can be clinically burdensome once they occur. A practical response would be to incorporate a brief oral-health screen at psychiatric admission, performed by the admitting team or another trained member of the inpatient team. That screen could include inspection for active caries, periodontal disease, facial swelling, or draining infection; review of medications that reduce salivary flow; assessment of tobacco and substance use; diabetes screening when indicated; and evaluation of the patient’s ability to cooperate with dental care. Patients with concerning findings would then follow a defined referral pathway to urgent dental or oral and maxillofacial surgery evaluation. The highest-yield group may be patients with repeated psychiatric admissions, documented substance use disorder, antipsychotic therapy, active dental disease, or facial swelling, although the present study cannot show that such a pathway would change outcomes.

### 4.1. Limitations

This study has several important limitations, and these limitations require the findings to be interpreted as exploratory. First, the denominator was returned by the institutional analytics tool as an aggregate count of unique psychiatric inpatients rather than as a complete patient-level follow-up file. The observed cases and the denominator were defined using the same Epic/SlicerDicer psychiatric-hospitalization criteria and the same study interval, so the observed cases met the eligibility definition for the denominator population. However, because the denominator was aggregate, we could not calculate dynamic follow-up time for every psychiatric inpatient from index psychiatric admission to death, loss to follow-up, or study end. Person-time was therefore approximated by assigning the full study duration to every patient in the denominator. This almost certainly overestimates true person-time; among cases with recoverable dates, the mean follow-up was approximately 5.7 years rather than 10.1. A shorter true person-time would lower the expected count and raise the observed-to-expected ratio.

Second, no demographic standardization was possible because stratified reference rates matching our cohort are not available and the denominator query did not provide a complete demographic file for the full psychiatric population. The psychiatric inpatient population differs from the general population in age, sex, race, socioeconomic position, tobacco and substance use, diabetes, and dental access. The ratio therefore reflects the combined burden of psychiatric hospitalization and these correlated exposures rather than an independent causal effect of psychiatric illness. Third, in 30 of 67 cases with sufficient timing data (45%), the abscess preceded the psychiatric admission. Psychiatric hospitalization therefore cannot have acted as an antecedent in those cases, which is why the result is framed as an association rather than an incidence estimate.

Fourth, the outcome definitions are not identical between our dataset and the published reference rates. Our cases were ascertained using K12.2 and J39.1, codes that denote infection already extending into soft tissue or fascial spaces. The published reference rates we applied are derived from national datasets coding periapical abscess (K04.6-K04.7), a more proximal entity that the ICD-10 structure does not place within K12.2 or J39.1. These populations overlap clinically, since a spreading odontogenic infection may carry both codes, but they are not the same disease denominator. The sensitivity analysis across reference rates addresses uncertainty in the magnitude of the reference rate but cannot resolve this mismatch in what is being counted. The direction of the resulting bias is not straightforward, and we do not claim to know it. In addition, the confidence intervals reflect Poisson variability in the observed count while treating the reference rate as fixed, so they understate total uncertainty.

Fifth, undercounting psychiatric admissions arriving by direct transfer would reduce the denominator and inflate the ratio, biasing the estimate away from the null. Finally, abstraction depended on documentation quality and, for some fields, varied among the several personnel who performed it; formal inter-rater agreement was not assessed. A common abstraction dataset was used, and discrepant or unclear entries were re-audited during revision using the frozen inclusion rule. Age and sex were complete for all 80 cases; race was missing in one; recurrence status was documented in 73; length of stay for the facial-abscess admission was documented in 76; psychiatric admission dates were recoverable in 67; cause of abscess was unspecified in 16; and anatomic space involvement was documented in nine. Other clinically relevant details, including source tooth, psychotropic medication class, ICU admission, airway intervention, microbiology, antibiotic management, and timing of dental follow-up, were not consistently captured and could not be reliably reported. As a single-center study, its generalizability beyond comparable safety-net settings is uncertain.

### 4.2. Future Directions

A future patient-level cohort study would be the most direct way to build on these findings. Such a study should define time zero at the first psychiatric admission, link the psychiatric population to incident facial-abscess events, and adjust for tobacco use, substance use, diabetes, socioeconomic position, and dental access. Prospective collection of involved fascial spaces, source teeth, psychotropic medication exposure, microbiology, antibiotic management, ICU admission, airway intervention, and dental follow-up would also help clarify the clinical pathway from oral disease to severe infection. Multicenter replication will be needed before the magnitude of the association can be generalized beyond safety-net hospitals similar to ours.

## 5. Conclusions

In this single-center safety-net dataset, patients with a documented history of psychiatric hospitalization were overrepresented among surgically managed facial abscess cases compared with what published population rates would predict. Because this is a crude observed-to-expected comparison without dynamic denominator-level person-time or demographic standardization, and because some abscesses preceded the psychiatric admission, the finding should be interpreted as an association reflecting psychiatric hospitalization and its accompanying exposures rather than as an independent causal effect. Even with those limitations, the study identifies a population in which severe facial infection is common enough to justify closer attention to oral health during psychiatric care. Patient-level linked cohort studies are needed to estimate risk more reliably.

## Figures and Tables

**Table 1:** Crude observed-to-expected ratio of surgically managed facial abscess in patients with a history of psychiatric hospitalization, across general-population reference rates. Observed cases = 80; person-time is approximate (see Methods). Expected cases are shown to two decimal places and ratios are computed from unrounded expected values. A higher reference rate yields a lower, more conservative ratio. CI, confidence interval; PY, person-years.

Reference Rate (per 100,000 PY)	Expected Cases	Ratio	95% CI	*p*-Value
2.0 (low reference rate)	2.69	29.7	23.6–37.0	<0.0001
2.5 (base case)	3.37	23.8	18.9–29.6	<0.0001
4.0 (high reference rate)	5.38	14.9	11.8–18.5	<0.0001

## Data Availability

The data presented in this study are available on request from the corresponding author. The data are not publicly available due to privacy and ethical restrictions on patient health information.
